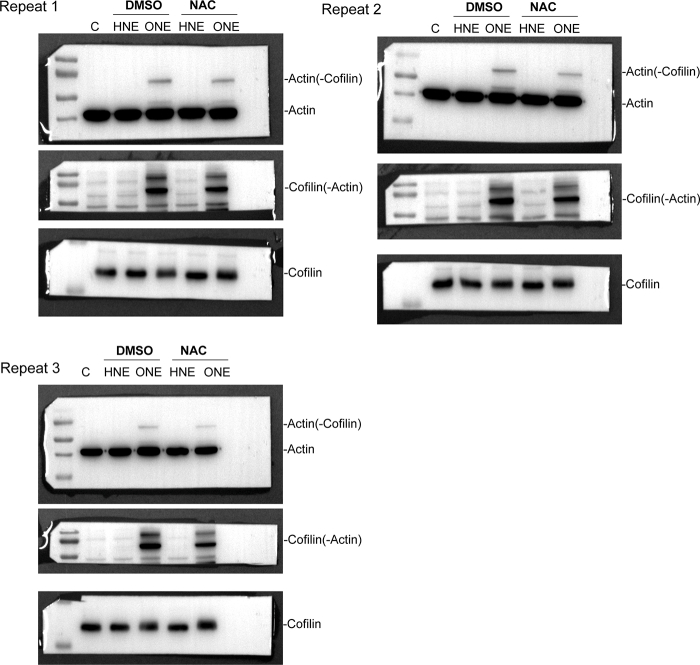# 4-Oxo-2-Nonenal Crosslinks Vicinal Cysteine-Lysine Pairs in Cellular Proteins

**DOI:** 10.1016/j.mcpro.2026.101573

**Published:** 2026-04-28

**Authors:** Ruifeng Cheng, Minran Wang, Keke Liu, Ling Fu, Ping Xu, Caiping Tian, Jing Yang

**Affiliations:** 1 School of Basic Medical Sciences, Anhui Medical University, Hefei, China; 2 State Key Laboratory of Proteomics, National Center for Protein Sciences Beijing, Beijing, China; 3 Beijing Key Laboratory of Environmental and Viral Oncology, College of Chemistry and Life Science, Beijing University of Technology, Beijing, China; 4 Guangzhou National Laboratory, Guangzhou International Bio-Island, Guangzhou, China

## Abstract

4-Oxo-2-nonenal (ONE) is a highly reactive aldehyde derived from endogenous lipid peroxidation, which is associated with the development and progression of chronic human diseases. Despite the fact that ONE exerts biological effects mainly by modifying nucleophilic amino acid residues on proteins in cells, whether and how this bifunctional electrophile forms covalent crosslinks in complex proteomes remains unknown. Here, we report a reanalysis of the data previously reported where *in situ* ONE adduction chemistry has been surveyed by chemoproteomics (Sun *et al*., *Mol Cell Proteomics*, 2017), revealing that vicinal Cys and Lys residues on cellular proteins can be directly crosslinked by ONE *via* initial Michael addition and subsequent Paal-Knorr condensation. Furthermore, we adapted our chemoproteomics platform to enable more efficient and robust crosslinking-mass spectrometry analysis, resulting in the identification of a total of 165 pyrrole-based Cys-Lys crosslinks on 145 proteins from intact cells. Quantitative analysis reveals the dynamic nature of these crosslinks *in situ*, which is similar to most ONE-derived modifications (*i.e*., lysine Schiff base adducts, lysine ketoamide adducts, and cysteine pyrrole adducts). Biochemical analyses further highlight the functional importance of one of the identified crosslinking events; an intermolecular crosslink between cofilin and actin proteins might partially be involved in apoptosis induced by this cytotoxic electrophile. Taken together, our study not only expands the inventory of ONE-adducts in cells, but also provides additional mechanistic insights into the potent cytotoxicity by this endogenous electrophile.

## Highlights

• First discovery of ONE-mediated Cys-Lys crosslinks in cells.
• 165 pyrrole-based crosslinks identified on 145 proteins.
• Dynamic turnover of crosslinks revealed by quantitative XL-MS.
• COF1-ACTG crosslink partially contributes to ONE-induced apoptosis.

## Graphical Abstract

**Figure:**
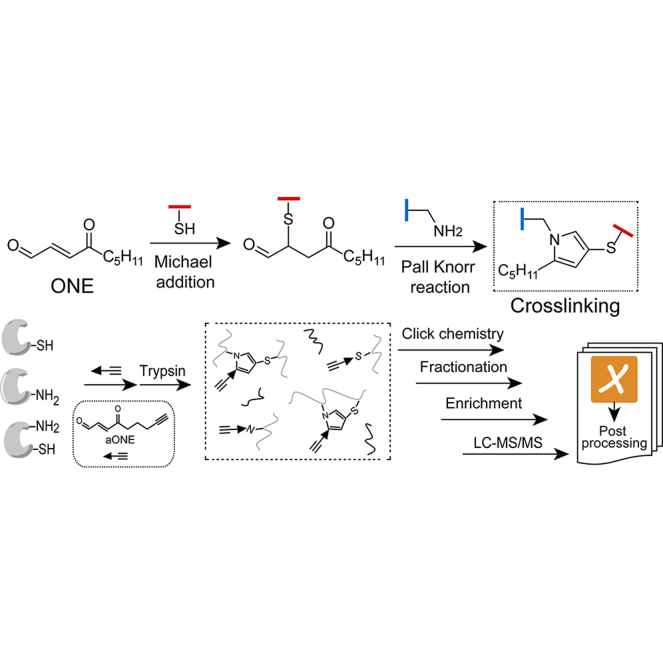

Polyunsaturated fatty acids (*e.g*., linoleic acid and linolenic acid) are susceptible to peroxidation under oxidative stress and produce many reactive carbon electrophiles (1). 4-oxo-2-nonenal (ONE, Fig. 1*A*), a product of lipid peroxidation first reported in 2000 (2), has been demonstrated to play important roles in diverse biological processes and cellular signaling, including oxidative stress adaptation, DNA damage, and epigenetic regulation (3, 4, 5). Elevated ONE levels have been linked to the progression of chronic human diseases such as neurodegenerative diseases and cardiovascular diseases (6, 7, 8).

**Fig. 1:**
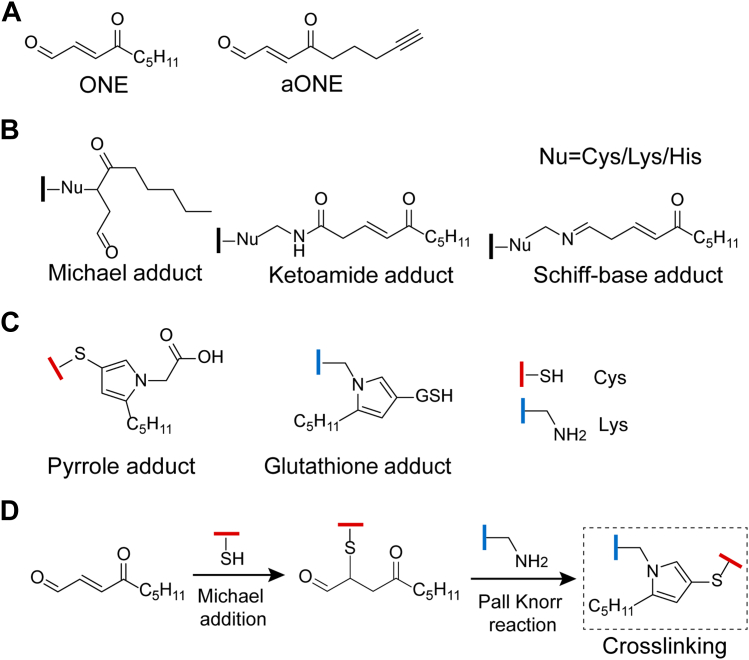
***In situ* adduction chemistry by 4-oxo-2-nonenal.***A*, chemical structure of ONE and its clickable analog aONE. *B*, chemical structure of ONE-mediated protein adduction on nucleophilic residues. *C*, chemical structure of ONE-mediated crosslinks between protein and small molecule metabolites. *D*, scheme of ONE-mediated protein crosslinking. aONE, alkynyl-ONE; ONE, 4-oxo-2-nonenal.

The biological activities of ONE are largely attributed to its potent electrophilicity. ONE contains an alpha, beta-unsaturated aldehyde group to rapidly react with the side chains of nucleophilic amino acid residues *via* Michael addition (Fig. 1*B*) (9, 10, 11, 12). ONE can also form reversible Schiff base adducts with Lys residues, followed by oxidation to more stable ketoamide adducts (Fig. 1*B*) (13, 14). Such adduction chemistry was initially characterized *in vitro* using model systems (*i.e*., small molecules or recombinant proteins), and could also be confirmed in complex biological systems (4, 15, 16).

In addition, it has been suggested that high concentrations (*e.g*., several millimolar) of ONE can induce substantial intra or intermolecular crosslinking of its targets *in vitro*. Such crosslinking events have also been associated with many disease proteins, such as pyruvate kinase M2, peptidylprolyl *cis/trans* isomerase A1 and synuclein (17, 18, 19). Interestingly, Sayre and coworkers found that ONE could form Lys-Lys, Cys-Lys and His-Lys crosslinks using model small molecules (9). Despite these interesting findings, whether and how ONE forms covalent crosslinks in native biological systems, especially in an intact cellular context, remains unexplored.

Using the combination of chemoproteomic profiling and blind search-based informatic analysis, we previously characterized *in situ* crosslinking of small molecule metabolites (*i.e*., GSH, glycine) to proteins by alkyne ONE (aONE, Fig. 1*A*), a well-established “clickable” surrogate of ONE (15, 20, 21, 22). Competitive assays further confirmed that aONE faithfully mimics ONE’s reactivity (Supplemental Fig. S1). Specifically, aONE initially reacts with protein cysteinyl thiols *via* Michael addition, followed by the formation of the pyrrole-based crosslink with the primary amine group of free glycine *via* the Paal Knorr reaction (Fig. 1*C*) (15). Alternatively, aONE first reacts with thiol-containing small molecules like GSH, followed by the formation of the pyrrole-based crosslink with lysine residues on proteins (Fig. 1*C*) (23). Inspired by these findings, we hypothesized that vicinal Cys and Lys residues on cellular proteins might also be directly crosslinked by ONE *via* initial Michael addition and subsequent Paal-Knorr condensation (Fig. 1*D*).

By reanalyzing the aONE-based chemoproteomic datasets previously reported (15), here we confirmed the hypothesis that ONE can mediate Cys-Lys crosslinking in cellular proteins. Furthermore, we adapted our chemoproteomics platform to enable more efficient crosslinking mass spectrometry (XL-MS) analysis, resulting in a total of 165 cross-linked intra and intermolecular Cys-Lys pairs in 145 proteins in RAW264.7 cells, which have also been used in other ONE studies (24, 25). Quantitative analysis reveals the dynamic nature of these crosslinks *in situ*, which is similar to most ONE-derived modifications (*i.e*., lysine Schiff base adducts, lysine ketoamide adducts, and cysteine pyrrole adducts). Biochemical analyses further showed that an intermolecular Cys-Lys crosslink between cofilin and actin proteins might contribute partially to ONE-induced apoptosis, underscoring the functional significance of this protein crosslinking event.

## Experimental Procedures

### Chemicals

Alkynyl-ONE (aONE, cat.no.17104) and ONE (cat.no.10185) were purchased from Cayman. 4-hydroxy-2-nonenal (cat.no. HY-113466) was purchased from MCE. N-acetylated cysteine (cat.no. S1623) was purchased from Selleck. 12C and 13C labeled Az-UV-biotin reagents were purchased from Kerafast (cat. no. EVU102 and EVU151). iodo-N-(prop-2-yn-1-yl) acetamide (IPM, cat.no. EVU111) and sulfotetrafluorophenyl (STP, cat.no. 30720) alkyne probes were purchased from KeraFast and Lumiprobe Corporation, separately. DTT (cat. no. A620058-0025) was purchased from BBI Life Sciences. HPLC-grade water, acetonitrile (ACN), and methanol (MeOH) were purchased from J. T. Baker. Other chemicals and reagents were obtained from Sigma-Aldrich unless otherwise indicated.

### Antibodies

Anti-Cofilin (GeneTex, GTX632582, diluted at 1:1000); anti-Actin (Abcam, ab179467, diluted at 1:1000); goat anti-mouse HRP (ZSGB-BIO, ZDR5307, diluted at 1:2500); goat anti rabbit-HRP (ZSGB-BIO, ZDR5306, diluted at 1:2500).

### Cell Culture

RAW264.7 and HEK293T cells from ATCC were cultured in Dulbecco's Modified Eagle Medium containing 10% (vol/vol) fetal bovine serum (Gibco, cat. no. C11995500), 1% (vol/vol) penicillin and streptomycin (Cell World, cat. no. C0160-611). Cells were maintained at 37 °C in a 5% CO_2_ humidified atmosphere

### Cell Treatment

RAW264.7 cells were grown to 80% confluence and incubated with 100 μM aONE for 2 h in serum-free medium. Treatments were terminated by removing the medium. Cells were then mechanically scratched and harvested by centrifugation at 1500 *g* for 3 min. For recovery experiments, cells were cultured as above, labeled for 2 h with 100 μM aONE, either harvested immediately (0 h) or recovered after 1 h and 4 h time points in serum-free medium without probe. For the detection of the COF1-ACTG complex, RAW264.7 cells were grown until 70 to 80% confluent in six well plates. After overnight serum deprivation, cells were incubated with indicated concentration of ONE in serum-free medium for up to 2 h at 37 °C. The concentration of ONE (100 μM) used in this study was selected based on established practices in the field, where exogenous concentrations ranging from 50 to 200 μM are commonly employed to study ONE-mediated protein modifications and cellular responses. Such concentrations have been well-documented to induce robust and reproducible biochemical phenotypes (15, 17, 24, 25). This concentration falls within the upper range of those used to elicit clear phenotypic readouts, enabling reliable detection of crosslinking events and facilitating subsequent mechanistic investigation.

### Sample Preparation for XL-MS Experiments

RAW246.7 cells treated with aONE were lysed on ice in lysis buffer (50 mM Hepes, 150 mM NaCl, 1% Igepal, pH 7.5) containing inhibitor cocktail. The lysate was incubated with 8 mM DTT at 75 °C for 15 min, followed by iodoacetamide (IAM)-based alkylation (32 mM, 30 min at room temperature (RT)). Proteins were then precipitated with MeOH-chloroform (aqueous phase/MeOH/chloroform, 4:4:1 (v/v/v)) as previously described (26). The precipitated protein pellets were resuspended with 50 mM ammonium bicarbonate containing 0.2 M urea. Resuspended proteins (2 mg/ml) were digested with sequencing grade trypsin (Promega) at a 1:50 (enzyme/substrate) ratio overnight at 37 °C. The tryptic digests were desalted with HLB SPE cartridges (Waters). The desalted tryptic digests were then evaporated to dryness under vacuum and then resuspended in an aqueous solution containing 30% ACN. Click chemistry was performed by the addition of 0.8 mM light Az-UV-biotin reagent, 8 mM sodium ascorbate, 1 mM TBTA, and 8 mM CuSO_4_. Samples were allowed to react at RT for 2 h with rotation and light protection. For recovery experiments, control (0 h) and recovery groups (1 h and 4 h) were reacted with 0.8 mM light and heavy Az-UV-biotin reagents, respectively, and combined equally after the click chemistry reaction. In brief, the sample was diluted into strong cation exchange (SCX) loading buffer (5 mM KH_2_PO_4_, 25% MeCN, and pH 3.0), passed through the SCX spin columns (The Nest Group) and washed with several column volumes of loading buffer. The retained peptides were eluted with a series of high-salt buffers containing 200 mM, 400 mM, 600 mM and 800 mM NaCl. Preliminary experimental results showed that the peptides eluted with 400 mM and 600 mM NaCl yielded the highest number of cross-linked peptides. We choose the peptides eluted with 400 mM and 600 mM NaCl.Then, they were combined and diluted 10× with 50 mM sodium acetate buffer (pH 4.5) and then subject to the enrichment with streptavidin agarose beads (GE). The elutes were allowed to incubate with prewashed streptavidin Sepharose for 2 h at RT. Streptavidin Sepharose then was washed with 50 mM NaOAc, 50 mM NaOAc containing 2 M NaCl, and water twice each with vortex and/or rotation to remove nonspecific binding peptides, and resuspended in 25 mM ammonium bicarbonate. The suspension of streptavidin Sepharose was transferred to glass tubes (VWR), irradiated with 365 nm UV light (Entela) for 2 h at RT with stirring. The supernatant was collected, evaporated to dryness under vacuum, and stored at −20 °C until analysis.

### Sample Preparation for Competitive ABPP Experiments

RAW246.7 cells (1∗10 cm dish) were cultured as above and treated with 50 μM ONE or vehicle (dimethyl sulfoxide (DMSO)) for 2 h. The cells were then lysed on ice in lysis buffer (50 mM Hepes, 150 mM NaCl, 1% Igepal, and pH 7.5) containing inhibitor cocktail. The lysates (∼1 mg protein) were equally divided into two aliquots and labeled with 100 μM IPM or 100 μM STP alkyne at RT for 1 h, respectively. The probe-labeled lysates incubated with 8 mM DTT at 75 °C for 15 min, followed by IAM-based alkylation (32 mM, 30 min at RT). Proteins were then precipitated with MeOH-chloroform (aqueous phase/MeOH/chloroform, 4:4:1 (v/v/v)) as previously described (26). The precipitated protein pellets were resuspended with 50 mM ammonium bicarbonate containing 0.2 M urea. Resuspended proteins (2 mg/ml) were digested with sequencing grade trypsin (Promega) at a 1:50 (enzyme/substrate) ratio overnight at 37 °C. The tryptic digests were desalted with HLB SPE cartridges (Waters). The desalted tryptic digests were then evaporated to dryness under vacuum and then resuspended in an aqueous solution containing 30% ACN. Click chemistry was performed by the addition of 0.8 mM either light (ONE treatment) or heavy (DMSO) Az-UV-biotin reagents, 8 mM sodium ascorbate, 1 mM TBTA, and 8 mM CuSO_4_. Samples were allowed to react at RT for 2 h with rotation and light protection. The light and heavy samples were then mixed together after the click chemistry reaction. Excess reactants were removed by SCX spin columns as previously described (26) and then the elutes were allowed to incubated with prewashed streptavidin Sepharose for 2 h at RT. Streptavidin Sepharose then was washed with 50 mM NaOAc, 50 mM NaOAc containing 2 M NaCl, and water twice each with vortex and/or rotation to remove nonspecific binding peptides, and resuspended in 25 mM ammonium bicarbonate. The suspension of streptavidin Sepharose was transferred to glass tubes (VWR), irradiated with 365 nm UV light (Entela) for 2 h at RT with stirring. The supernatant was collected, evaporated to dryness under vacuum, and stored at −20 °C until analysis.

### LC-MS/MS Analysis

The LC-MS/MS analysis was performed using a Orbitrap Q Exactive HF-X mass spectrometer coupled with an UltiMate 3000 LC system (Thermo Fisher Scientific). A 15-cm-long LC column (i.d. 150 μm) packed with 1.9 μm C18 packing particles were used for peptide separation. The column was pulled using a micropipette puller (P-2000, Sutter Instrument) for preparation of the nanoESI tips with a ∼5 μm opening. The spray voltage was set at 2.3 kV. An 80-min gradient of 6 %-40% buffer B (80% ACN with 0.1% formic acid) at a flow rate of 600 nl/min was used for peptide elution. High energy collisional dissociation (HCD) MS/MS spectra were recorded in the data-dependent mode using a top 25 method. MS1 spectra were measured with a resolution of 120,000 (at *m/z* 200), an AGC target of 3e6, a max injection time of 20 ms, and a mass range from *m/z* 350 to 1550. HCD MS/MS spectra were acquired with a resolution of 15,000, an AGC target of 2e4, a max injection time of 30 ms, a 1.6 *m/z* isolation window and normalized collision energy of 27. For peptides from XL-MS experiments, divalent ions were excluded during MS/MS data acquisition. Charge states 3 to 8 were set for MS/MS scans. Peptide *m/z* that triggered MS/MS scans were dynamically excluded from further MS/MS scans for 18 s. For peptides from competitive activity-based protein profiling experiments, Charge states 1 to 8 were set for MS/MS scans. Peptide *m/z* that triggered MS/MS scans were dynamically excluded from further MS/MS scans for 15 s.

### pLink Search

Raw data files were searched against the mouse protein database downloaded from UniProt (reviewed, 17085 Uniprot ID included,download date 20211224) using pLink (version 2.3.9) (27, 28). Precursor ion mass and fragmentation tolerance were set as ±10 p.p.m. and ±20 p.p.m. Cysteine and lysine were set as crosslinking sites with the aONE-derived crosslinker mass of 271.132 Da. The maximum number of missed tryptic cleavages allowed was three. The peptide length and mass were allowed within the range of 6 to 60 amino acids and 600 to 6000 Da, respectively. Modifications of 15.9949 Da (Methionine oxidation, M) and +57.0214 Da (IAM alkylation, C) were searched as variable modifications. false discovery rate was set to ≤5% at the peptide-spectrum match level. Additional filtering criteria were applied; spectral count ≥3, E-value ≤1∗10^–5^. MS2 spectra were annotated using pLabel (29). The MS1-based quantification was performed using pQuant (30, 31), which calculates isotopic ratios based on each identified MS scan with a 15 ppm-level *m/z* tolerance window and assigns an interference score (σ) to each value from zero to one. The median values of probe-modified peptide ratios with σ less than or equal 0.5 were considered to calculate site-level ratios. Those aONE-crosslinked peptide assignments whose MS1 full scan reflected an invalid or not-a-number light/heavy ratio were discarded for following analysis. Quantification results were obtained from three biological replicates with single LC-MS/MS runs for each. The “Top25” precursors was selected for MS2 analysis, in which precursors were fragmented by HCD prior to Orbitrap analysis ((N)CE 27, AGC target 2 × 104, maximum injection time 30 ms, resolution 15,000, and isolation window: 1.6 Da).

### pFind Search

For the identification of aONE-derived modifications, data analysis was performed using the pFind studio (version 2.3.9) (32). Precursor ion mass and fragmentation tolerance were set as ±10 p.p.m. and ±20 p.p.m., respectively. The maximum number of modifications allowed per peptide was three, as was the maximum number of missed tryptic cleavages allowed. Modifications of 15.9949 Da (Methionine oxidation, M), +57.0214 Da (IAM alkylation, C), +289.1426 (C15H19N3O3, Schiff-base adduct, K), +307.1532 (C15H21N3O4, ketoamide adduct, K), +578.2159 (pyrrole-based GSH adduct, K) +311.1845 (C15H25N3O4, Michael adduct, C), +346.1641 (C17H22N4O4, pyrrole-based glycine adduct, C), were searched as variable modifications. No fixed modifications were searched. The maximum number of modifications allowed per peptide was three. A differential modification of 6.020132 Da on probe-derived modifications was used for stable-isotopic quantification. The false-discovery rates at spectrum, peptide, and protein level were ≤1%. The MS1-based quantification was performed using pQuant as above. Quantification results were obtained from three biological replicates with single LC-MS/MS runs for each.

### Transfection and Expression

pcDNA3.1-3x *Flag*-*CFL1* and C139A mutant were generated as previously described (15). Transfection was performed by incubating 4 μg of each of plasmids and 8 μl of ExFect transfection reagent (Vazyme Biotech CO., Ltd, T101-01) into 60% confluent HEK-293T cells on a 4 cm plate. Cells were cultured in Dulbecco's Modified Eagle Medium supplemented with 10% fetal bovine serum for another 24 h.

### Western Blot

Proteins from cell lysates were resolved by 12% SDS-PAGE and transferred to polyvinylidene difluoride (PVDF) membranes (Merck Millipore, IPVH00010). After transfer, the PVDF membrane was blocked with 5% milk in tris-buffered saline plus 0.05% Tween-20 (TBST) at RT for 1 h and incubated with the indicated primary antibodies overnight at 4 °C. After incubation, the membranes were washed three times with TBST and incubated with the corresponding HRP-conjugated specific secondary antibody. The PVDF membranes were then washed three times with TBST, and visualized on a Tanon 5200 scanner, and analyzed with GelCap software (version 5.6) using ECL chemiluminescence (CWBIO, CW0049S).

### In Gel Digestion

Protein lysates obtained from RAW246.7 cells treated with ONE were separated with SDS-PAGE. Gel bands corresponding to molecule weight (MW) 60 Kd were sliced off, transferred to microcentrifuge tubes, and destained with 50 mM of NH_4_HCO_3_ in 40% MeOH, followed by washed once with 50% and 75% ACN, then washed twice with water and 50 mM NH_4_HCO_3_, and then subjected to in gel trypsin digestion as previously describe (33). The resulting peptides were extracted, desalted, evaporated to dryness, and analyzed by LC-MS/MS.

### Annexin V/PI Kit

Annexin V, a Ca^2+^-dependent phospholipid-binding protein with a MW of 35 to 36 kDa, exhibits high-affinity binding to phosphatidylserine. During early apoptosis, phosphatidylserine—normally localized to the inner leaflet of the plasma membrane lipid bilayer—translocates to the outer leaflet. Fluorescently labeled Annexin V serves as a molecular probe, combined with membrane-impermeant nucleic acid dyes such as propidium iodide (PI) to distinguish viable cells (Annexin V−/PI−), early apoptotic cells (Annexin V+/PI−), and late apoptotic/necrotic cells (Annexin V+/PI+).

### Flow Cytometry-based Apoptosis Assay

Cell apoptosis was determined by flow cytometry using Annexin V and PI double staining. After treatment, cells were harvested with trypsin (without EDTA) and washed twice with cold PBS. According to the manufacturer’s protocol (Annexin V-FITC/PI Apoptosis Detection Kit, MCE, HY-K1073), approximately 1 × 10^5^ cells were resuspended in 200 μl of 1× binding buffer, followed by incubation with 5 μl of Annexin V-FITC and 10 μl of PI solution for 20 min at RT in the dark and then analyzed by BD Verse Flow Cytometry. Data analysis was conducted using FlowJo_10.8.1 software.

### Apoptosis Assay Data Analysis

The cells were classified as viable (Annexin V−/PI−), early apoptotic (Annexin V+/PI−), or late apoptotic/necrotic (Annexin V+/PI+). To minimize potential interference from other factors within the same experimental system, a relative quantification approach was adopted. Specifically, the apoptotic rate of each experimental group was normalized to its respective vehicle (DMSO) control. The relative apoptotic state was then calculated as the ratio of the apoptotic proportion after ONE treatment to that after DMSO treatment within the same cell group. Each experiment was performed in triplicate and repeated three times independently.

### Flow Cytometry for ROS Assays

RAW246.7 cells were seeded and treated with vehicle control, ONE (100 μM) and positive control (5 μM) for 2 h. After a 2 h incubation at 37 °C, cells were washed with PBS and stained with 10 μM dihydroethidium (SolarbioLife Sciences) for 30 min. Then the cells were harvested and analyzed by BD LSRFortessa SORP (BD Science). The data obtained was analyzed using FlowJo_v10.8.1 software.

### Experimental Design and Statistical Rationale

Cellular experiments were conducted with three biological replicates (independently cultured cells). "Triplicate biological replicates (independently processed samples) with duplicate technical measurements were implemented in proteomic sample preparation. Such design ensures: (a) Reduction of batch effects through biological replication; (b) Assessment of technical precision *via* repeated injections;(c) Sufficient statistical power (F-test df = 2, α = 0.05) for LC-MS/MS-based differential expression analysis.

### Statistical Analysis

Data are shown as mean ± SEM. *p* values were calculated using unpaired, two-tailed Student’s *t* test. *p* values of ≤0.05 were considered significant.

## Results

### Characterization of aONE-Derived Cys-Lys Crosslinks in Proteins

**Figure S2:**
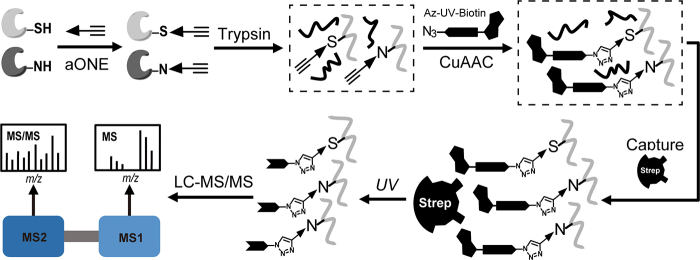


**Figure S3:**
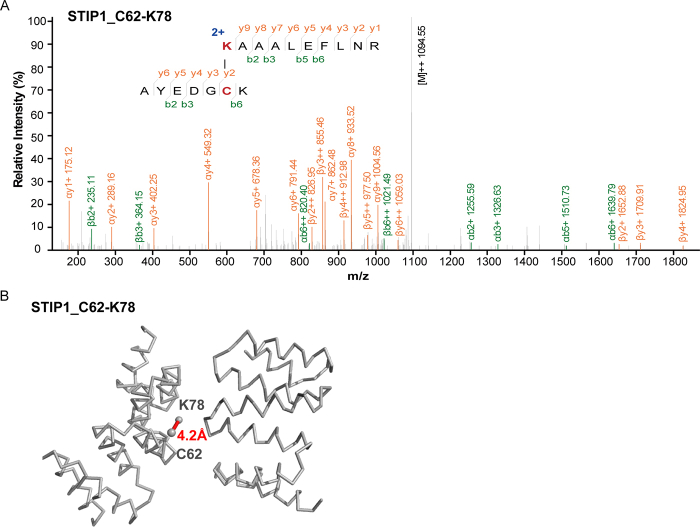


**Figure S4:**
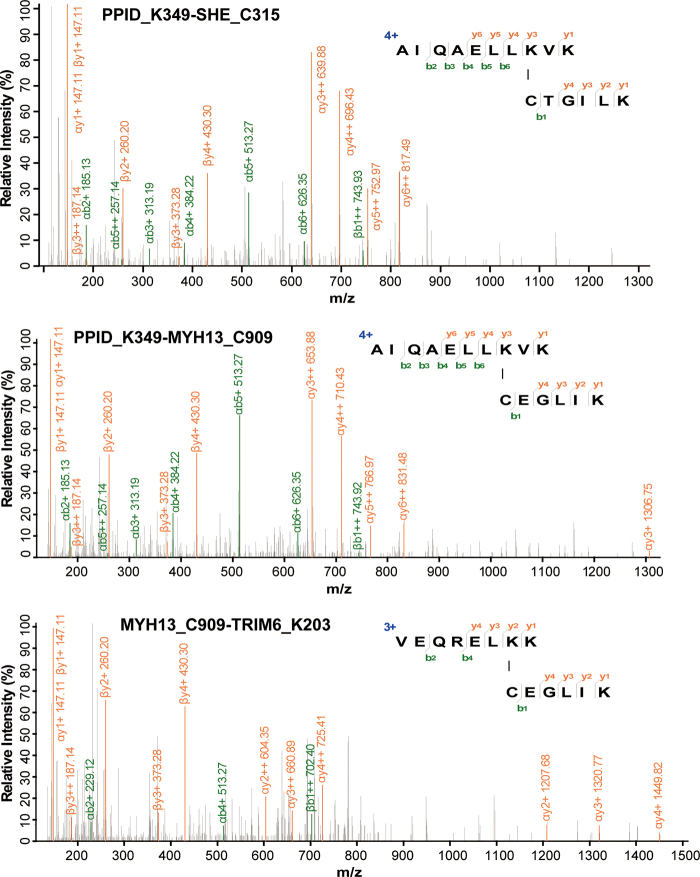


In our previous effort to investigate the adduction chemistry of ONE in native proteomes, we treated RKO cells with the aONE probe *in situ* (15). Then, the probe-treated proteome samples were harvested, processed by a well-established chemoproteomic workflow, and analyzed by LC-MS/MS (Supplemental Fig. S2) (26). The analysis allowed us to identify four types of probe-derived modifications, including Schiff-base and ketoamide adducts to lysine, Michael addition adducts to cysteine, and a novel pyrrole adduct to cysteine (15). To investigate whether aONE mediates the aforementioned pyrrole-based Cys-Lys crosslinks *in situ*, we reanalyzed the same MS data using pLink (28). Interestingly, we identified a total of 20 aONE-mediated cross-linked peptides and 45 loop-linked peptides, accounting for 0.93% and 2.09% of all identified peptides, respectively (Supplemental Table S1), while the identification rates of such crosslinked peptides were significantly lower than those of aONE-modified peptides (14.0%). As expected, the identified crosslinks could be mapped onto vicinal Cys-Lys pairs on proteins with recorded 3D structures. For example, a cross-link between Cys-62 and Lys-78 was identified within STIP1 protein (Supplemental Fig. S3*A*), whose spatial distance was 4.2 Å according to the 3D structure of this protein (PDB#: 1ELW, Supplemental Fig. S3*B*). Moreover, we found a number of crosslinking events across two different proteins. For example, PPID_K349 can form a cross-link with SEH1_C315 and MYH13_C909, respectively, while MYH13_C909 can form a crosslink with TRIM6_C203, which suggested the complex networks of protein cross-links mediated by ONE (Supplemental Fig. S4). Taken together, our initial attempt revealed that aONE can indeed mediate Cys-Lys crosslinking of cellular proteins, though the data were generated by experimental settings (*i.e*., sample preparation and MS analysis) that were not designed for identifying crosslinked peptides.

### Chemoproteomic Profiling of Protein Crosslinks Induced by aONE

**Fig. 2:**
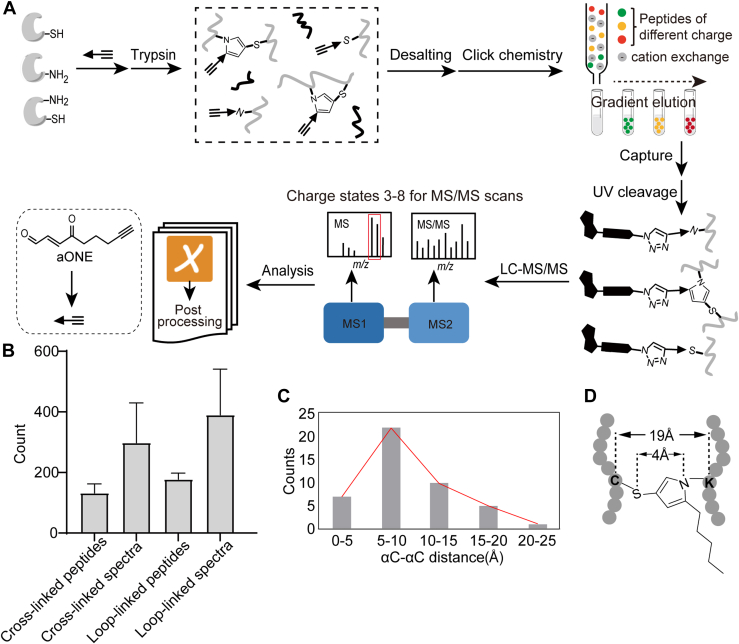
**Global profiling of aONE-derived protein crosslinks in cells.***A*, the optimized workflow for XL-MS analysis of aONE-derived protein crosslinks (See Methods for details). *B*, bar chart showing the numbers of aONE cross-linking peptides and the corresponding spectra identified in this study. Data are presented as the mean ± standard deviation (SD) from three independent experimental replicates (n = 3). *C*, bar chart showing the distribution of aONE cross-linking distances. *D*, the arm length of ONE-mediated crosslink. aONE, alkynyl-ONE; XL-MS, crosslinking mass spectrometry.

**Figure S5:**
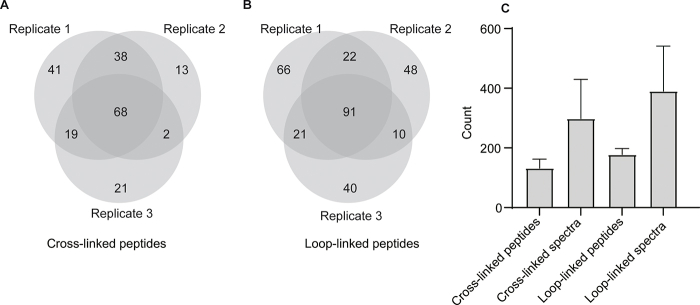


Encouraged by the aforementioned results, we sought to optimize several analytical parameters to facilitate the identification of crosslinked peptides (Fig. 2*A*). Considering that crosslinked peptides generally showed a higher charge (usually higher than positive divalent) than noncrosslinked peptides (34), we employed a stepwise salt gradient (0.2 M, 0.4 M, 0.6 M and 0.8 M NaCl) during SCX enrichment, pooling fractions eluted with 0.4 M and 0.6 M NaCl for subsequent streptavidin affinity purification. As in most XL-MS analyses, divalent ions were excluded during MS/MS data acquisition. Then we applied the optimized workflow to RAW264.7 cells treated with 100 μM aONE for 2 h *in situ* to profile Cys-Lys crosslinks across the proteome. Note that the probe concentration was chosen empirically based on previously published reports for the sake of consistency (15, 20). Probe-labeled protein samples were processed into tryptic peptides, “clicked” with Az-UV-biotin, enriched with streptavidin and photoreleased. The resulting peptides were subjected to LC-MS/MS analysis. MS data were searched against the corresponding protein database using pLink (28). To ensure the reliable identification of crosslinked peptides, we set up criteria as follows: false discovery rate ≤5%, spectral count ≥3, E-value ≤1e-5. Notably, 53% of cross-linked peptides and 51% of loop-linked peptides were commonly identified in all three biological replicates (Supplemental Fig. S5, *A* and *B*). Given the inherent complexity of crosslinked peptide identification and the stringent filtering criteria applied, these overlap rates demonstrate a high degree of experimental reproducibility. Overall, we identified 165 high-confidence crosslinked peptides from 145 unique proteins. The identification rates for cross-linked peptides (202) and loop-linked peptides (304) were 15.0% and 22.6% (Fig. 2*B*, Supplemental Fig. S5*C*, Supplementary Table S2), which were significantly higher than those obtained from nonoptimized workflow.

Next, we visualized 45 of the *in situ* detected crosslinks on the corresponding proteins with available 3D structures (Supplementary Table S3). Notably, 98% of the crosslinks were within the maximum distance constraint of 19 Å imposed by aONE. Moreover, the median values of αC-αC cross-linking distance were calculated to be 8.7 Å (ranging from 3.8 Å to 22.8 Å, Fig. 2*C*), which is even shorter than that (12.5 Å) obtained by the so-called “zero-length crosslinker” 1,1′-carbonyldiimidazole which has the crosslinker arm length of 2.6 Å (35). It is not unexpected as, theoretically, the five-member pyrrole ring formed by the ONE-mediated crosslinking is relatively rigid (4.0 Å of the crosslinker space arm plus 15 Å of protein flexibility, Fig. 2*D*), thereby rendering more constrainted crosslinking distances.

### Co-occurrence of aONE-Derived Modification and Crosslinking Events

**Figure S6:**
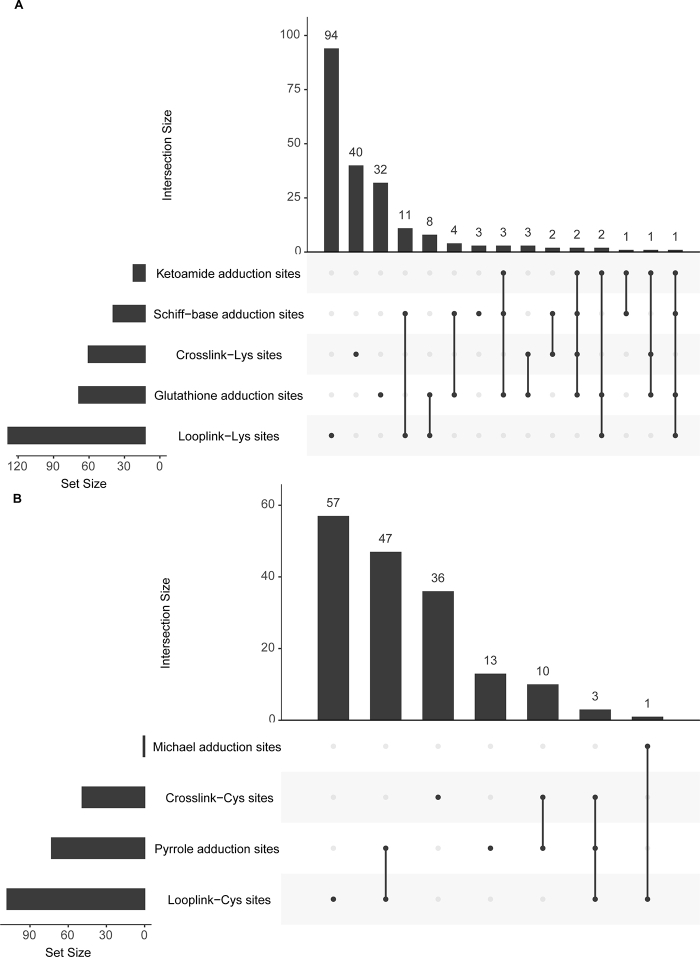


Given the shared electrophilic reactivity between ONE and its clickable surrogate aONE, it is not surprising that many of the Cys-Lys crosslinked sites identified using aONE overlapped with aONE-derived mono-adduction sites from our previous chemoproteomic profiling (15). For instance, residues such as PRDX1_C173 (pyrrole adduct), HNRPL_K490 (ketoamide adduct), HDGF_K106 (Schiff-base adduct), PRDX1_K178 (GSH adduct), and IF5_C59 (Michael adduct) were all identified as sites of aONE mono-modification, and were also found to participate in aONE-mediated crosslinking events (Supplementary Table S4). This co-occurrence supports the notion that these vicinal Cys-Lys pairs may serve as privileged sites for ONE-induced covalent crosslinking. We further compared the crosslinked sites identified in this study with the aONE-derived mono-adducts and loop-linked sites profiled in our dataset (Supplemental Fig. S6 and Supplementary Table S2). Notably, the overlap between crosslinked sites and mono-modified or loop-linked sites is relatively limited. For example, only a small fraction of cysteine residues involved in crosslinking were also found to be mono-adducted or loop-linked. This observation suggests a degree of specificity in the formation of crosslinks, although we acknowledge that the limited overlap may also be partially attributed to the current profiling depth, as crosslinked peptides are inherently more challenging to detect and quantify than mono-modified peptides.

**Fig. 3:**
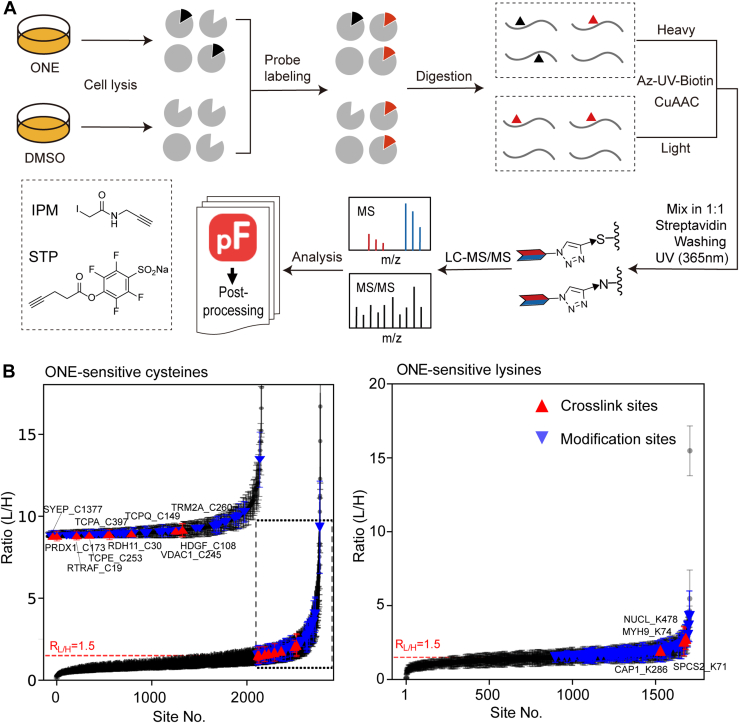
**Competitive profiling of ONE-sensitive sites in the proteome.***A*, workflow for competitive experiments. Competitive analysis of the RAW246.7 cell proteome pre-treated with ONE (100 μM, 2h) or DMSO vehicle, followed by a probe that is broadly reactive for cysteines (IPM) or lysine (STP). Sites were designated as ONE-sensitive if the R_L/H_ (DMSO *versus* ONE) value was ≥1.5. *B*, rank plot of R_L/H_ values comparing DMSO- *versus* ONE-treated samples. *Red* and *blue dots* represent aONE-derived crosslinking sites and modification sites, respectively. Data represent the mean ± SD from three independent experimental replicates. aONE, alkynyl-ONE; DMSO, dimethyl sulfoxide; IPM, iodo-N-(prop-2-yn-1-yl) acetamide; ONE, 4-oxo-2-nonenal.

To further delineate the relationship between the residue-oriented reactivity of the crosslinking site, we adapted a competitive activity-based protein profiling method which has been the method of choice for mapping targeted sites of electrophile-derived covalent modification (36, 37, 38, 39, 40, 41, 42, 43, 44, 45) to globally quantify the parent compound ONE against proteomic cysteines and lysines, respectively (Fig. 3*A*). Specifically, RAW264.7 cells were incubated with 100 μM ONE or DMSO for 2 h and then harvested. The cell lysates treated with or without ONE were reacted with the cysteine-specific probe called IPM (26) or the lysine-specific probe called STP alkyne (46), and then digested into tryptic peptides. Probe tagged peptides were then ‘clicked’, cleaned with SCX, enriched with streptavidin, photo-released and analyzed with LC-MS/MS. The light/heavy (L/H = control/treatment) ratio calculated for each probe-labeled site in the proteome provided a measure of its relative reactivity in controls *versus* ONE-treated samples. In principle, modification or crosslinking of a site should reduce its accessibility to the corresponding probes. Hence, the higher the measured competitive ratio for a quantified site, the more likely it was a target in this analysis. Three biological replicates with one technical LC-MS/MS run were performed for each experiment, resulting in six high-resolution MS/MS datasets.

**Figure S7:**
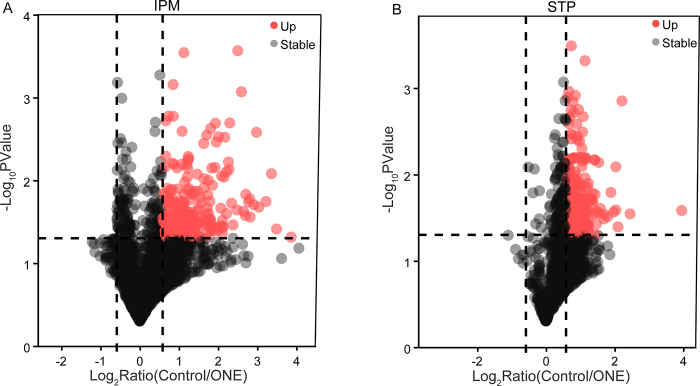


**Figure S8:**
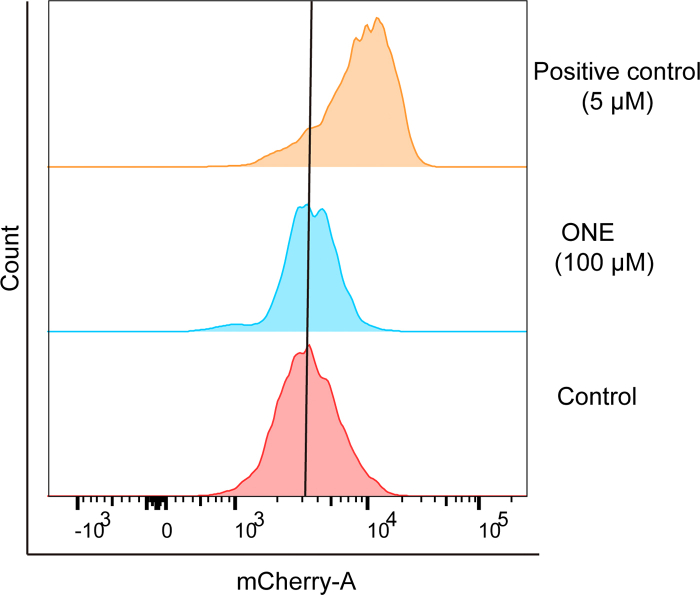


In total, we quantified 2772 IPM-labeled cysteine sites on 1944 proteins and 1706 STP alkyne-labeled lysine sites on 1043 proteins with a CV value < 40% for each site (Supplementary Table S5). Of note, 24% (675 in 2772) of quantified IPM-labeled cysteines and 48% (811 in 1706) of quantified STP alkyne-labeled lysines showed R_L/H_ ≥ 1.5 (*i.e*., fold-change higher than 1.5) after ONE treatment, in accordance with the high reactivity of this potent electrophile (Fig. 3*B*). Among these specific sites competitively labeled by IPM and STP, 261 and 323 sites showed both quantitative and statistical differences, respectively (Supplemental Fig. S7, *A* and *B*). Considering that electrophilic small molecules likely mediate changes in cellular oxidative state, we investigated whether ONE induced changes in ROS levels in cells. However, ONE did not mediate an increase in ROS levels in cells (Supplemental Fig. S8). This result indicates that the reduction in labeling is caused by direct competition, rather than changes in cysteine oxidation states. While our ROS measurements suggest minimal oxidative interference, changes in probe labeling could theoretically also arise from other indirect effects such as steric hindrance or altered protein conformation. Regardless, 10 cysteine and four lysine sites were also involved in the aONE mediated crosslinking events; 53 cysteines and 96 lysines were identified as the aONE-modified sites (Fig. 3*B* and Supplementary Table S5).

### Dynamics of aONE-Derived Crosslinks in Cells

**Fig. 4:**
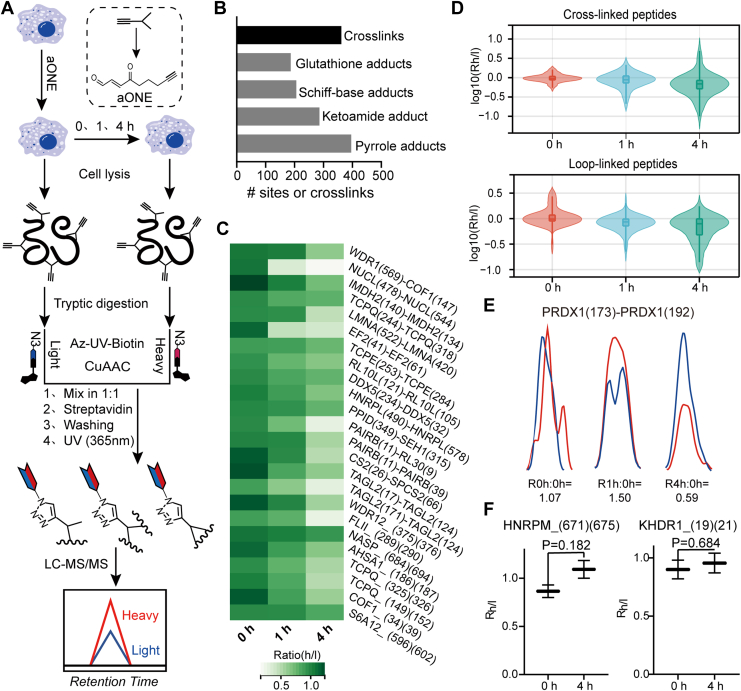
**Dynamic of aONE-derived protein crosslinks in cells.***A*, workflow for quantitative chemoproteomic analysis of aONE-derived protein modifications and crosslinks. RAW246.7 cells were first treated with 100 μM aONE. After 2 h incubation, cells were either harvested immediately and used as controls or placed in aONE-free medium for another 1 and 4 h recovery period. The aONE-labeled proteomes were digested with trypsin and then biotinylated by click chemistry with the light (control) or heavy (recovery) labeled UV-cleavable azido biotin, followed by streptavidin enrichment, photorelease, and LC-MS/MS based identification and quantification. *B*, bar chart showing the numbers of aONE-derived protein crosslinks and modifications identified in this study. Data from three independent experimental replicates (n = 3). *C*, heatmap showing the quantified ratios of aONE-derived crosslinks on cellular proteins over the indicated recovery period. The ratios were derived from three independent experimental replicates. *D*, quantitative changes of an aONE crosslinked peptide from PRDX1 over time. Data represent the mean ± SD from three independent experimental replicates *E*, representative XIC chromatogram showing the profiles for light- and heavy-labeled peptides (in *blue* and *red*, respectively) for the aONE-derived crosslink to PRDX1 (C173-K192). Similar results were obtained from three independent experimental replicates. *F*, box plots showing two representative aONE crosslinks with relatively high stability in cells. aONE, alkynyl-ONE.

In our previous report (15), we set up a recovery experiment to measure the dynamics of aONE-derived modifications in cells. This analysis revealed fast *in situ* turnover rates of most ONE-mediated protein modifications such as Michael addition and glycine-derived pyrrole adducts on cysteine, Schiff base and ketoamide adducts on lysine. In contrast, GSH-aONE-lysine conjugates showed a high stability in cells during the recovery period of 4 h (23). Nonetheless, it is not clear whether ONE-mediated protein crosslinks are also subject to dynamic changes in the same condition. In the same design of recovery experiment, the optimized XL-MS workflow (Fig. 2*A*) was applied to ratiometrically quantify dynamic changes in aONE-derived Cys-Lys crosslinks in cells. Specifically, RAW264.7 cells were incubated with 100 μM aONE for 2 h and then harvested immediately (as controls) or placed in fresh culture medium and allowed to recover for another 0 h, 1 h and 4 h, respectively. Proteins obtained from each group were then digested into tryptic peptides and conjugated with light or heavy labeled UV-cleavable azido biotin *via* click chemistry, followed by streptavidin enrichment, photorelease, and LC-MS/MS analysis (Fig. 4*A*). Quantification was achieved with the use of isotopically encoded light and heavy signals on the “clicked” aONE-modified and crosslinked peptides across three biological replicates as aforementioned.

**Figure S9:**
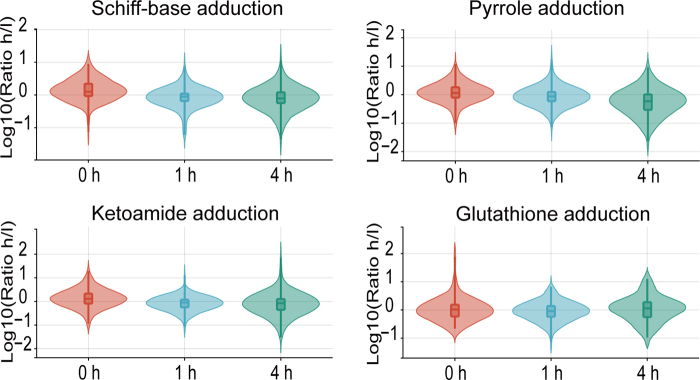


**Figure S10:**
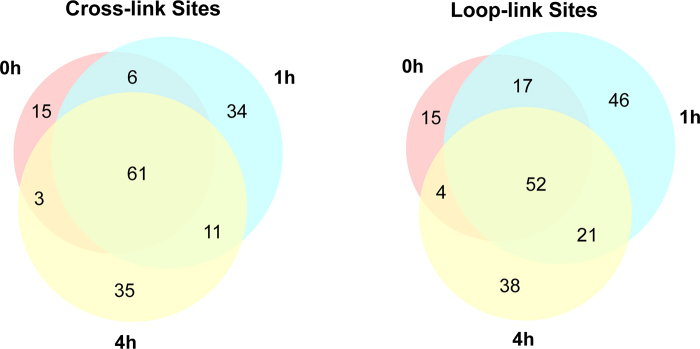


Overall, we simultaneously identified 286 lysine ketoamide adducts, 206 lysine Schiff-base adducts, 187 lysine GSH adducts, 396 cysteine pyrrole adducts and 362 pyrrole-based Cys-Lys crosslinks (Fig. 4*B* and Supplementary Table S6). The data not only confirmed our previous findings in terms of the turnover rates of all detected aONE modifications in cells (Supplemental Fig. S9), but also allowed us to quantitatively measure 61 cross-linked and 52 loop-linked Cys-Lys pairs on 49 proteins in all three recovery periods (Fig. 4*C*, Supplemental Fig. S10 and Supplementary Table S6). In general, only ∼26% of aONE crosslinking events showed at least a 1.5-fold decrease at 1 h of recovery, whereas such percentage dramatically increased to ∼45% over the course of 4 h (Fig. 4*D*). For example, we observed a significant loss of crosslink on PRDX1_K192-C173 in a 4 h recovery period(R_h/l_ = 0.75, Fig. 4*E*). Nonetheless, similar to aONE modifications, crosslinking events mediated by this electrophile also showed the site-specific stability. For example, we found that a few aONE-crosslinked Cys-Lys pairs (*e.g*., HNRPM_K671-C675, KHDR1_C19_K21) remained quite stable at 4 h of recovery (Fig. 4*F*), indicating more potent and long-lived biological effects of these crosslinking events. It should be noted that the observed decay in crosslinked signals may reflect not only the intrinsic stability of the crosslink itself, but also the turnover of the parent proteins. Distinguishing between these contributions would require additional orthogonal approaches, such as pulse-chase labeling, which merits future investigations.

### Functional Analysis of ONE-Derived Crosslinks

**Figure S11:**
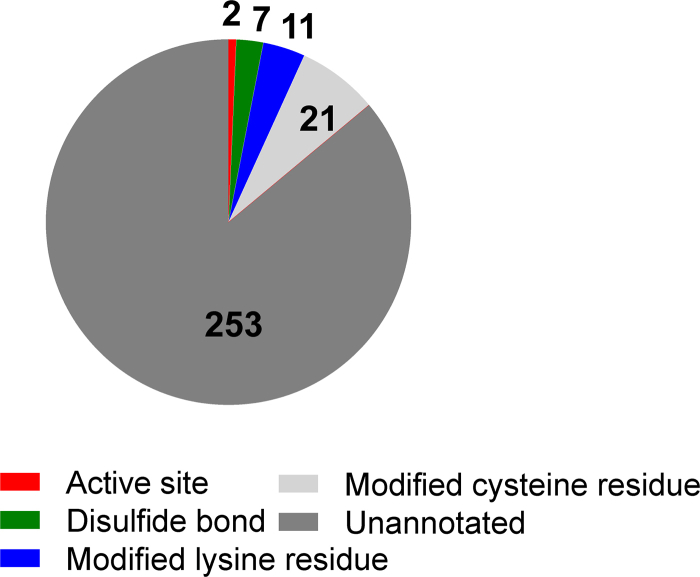


**Figure S12:**
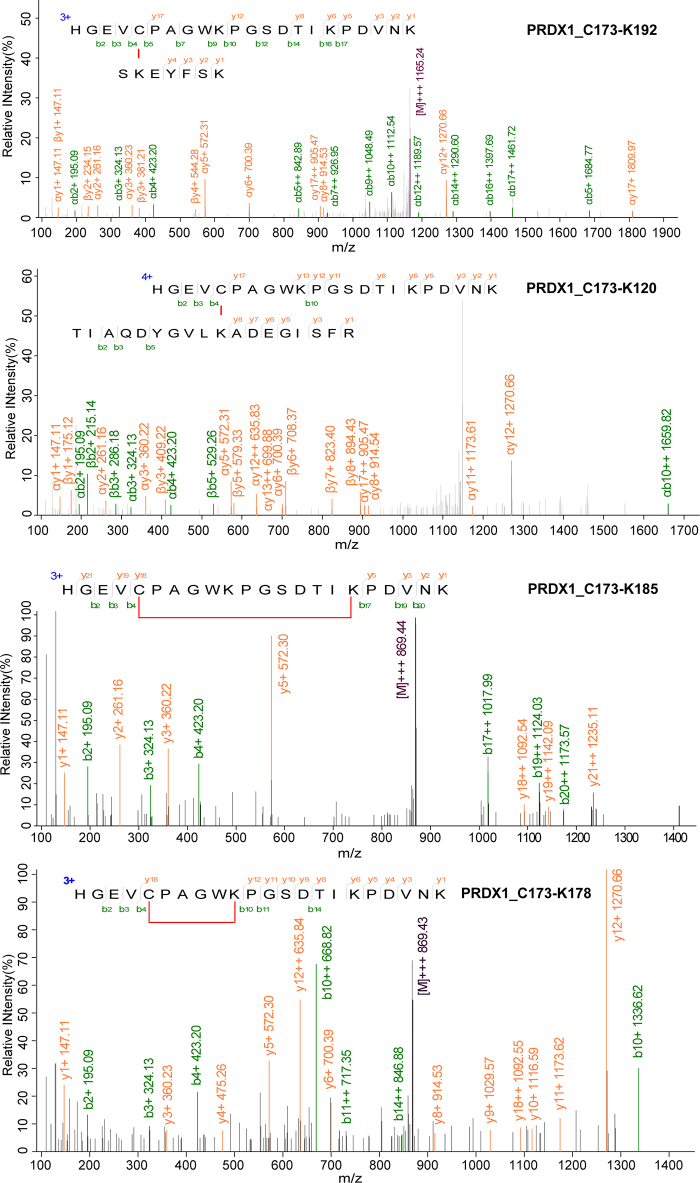


Overall, our analyses identified a total of 165 aONE-derived Cys-Lys crosslinks on 145 cellular proteins, while the functional impact of these *in situ* crosslinking events remained to be determined. Interestingly, we found many of these crosslinks occured on functionally important sites, including several well-characterized redox-regulated cysteine residues such as the peroxiredoxins (Supplemental Fig. S11) (47, 48, 49, 50). For example, the conservative resolving cysteine Cys 173 forms inter-crosslinks by aONE with its neighboring lysine residues, including K120, K178, K185 and K192 (Supplemental Fig. S12). Note that Cys 173 of PRDX1 is critical for the restoration of its enzymatic activity *via* the reversible disulfide bond formation with the catalytic site Cys 52 (*i.e*., peroxidatic cysteine), thereby transducing redox signaling (51). Hence, our results further highlight the potential crosstalk among ONE-mediated protein Cys-Lys crosslinks and redox thiol modifications.

**Fig. 5:**
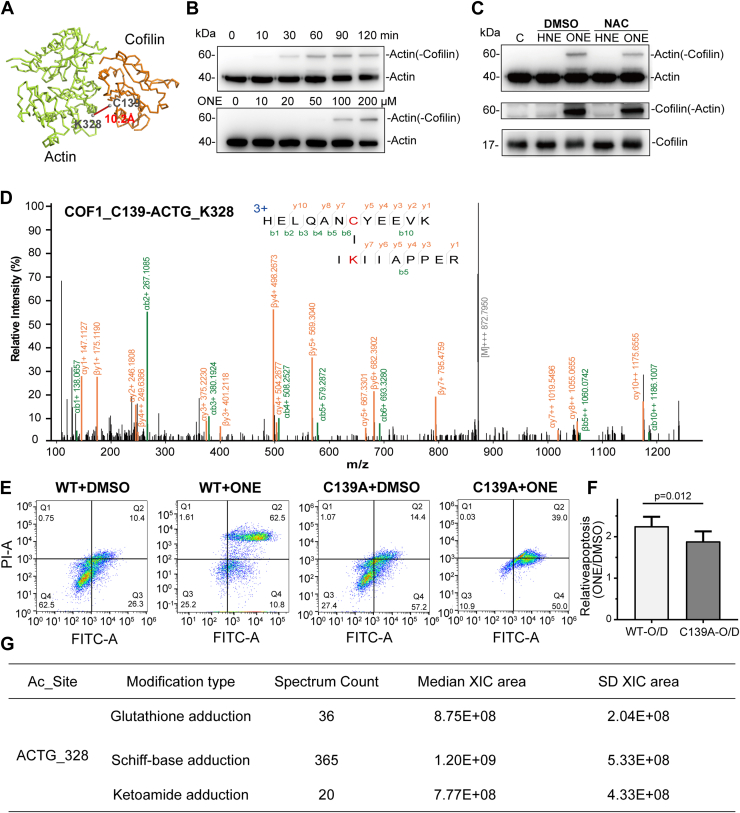
**Functional analysis of an ONE-derived actin-cofilin crosslink.***A*, 3D structure (6VAO) of the actin-cofilin complex on which a ONE-crosslinked sites mapped on their interacting interface. *B*, RAW246.7 cells were treated with ONE at indicated time (*upper*) and concentration (*lower*). After 2 h incubation, cells were harvested to yield lysates to be resolved by SDS-PAGE and subjected to Western blotting with indicated antibodies. *C*, RAW246.7 cells were then treated with ONE or HNE in the presence and absence of NAC, respectively. After 2 h incubation, cells were harvested to yield lysates to be resolved by SDS-PAGE and subjected to Western blotting with indicated antibodies. *D*, representative MS/MS spectrum of the ONE-crosslinked ACTG(K326)-COF1(C139) peptide. Protein lysates obtained from RAW246.7 cells treated with ONE (100 μM, 2 h) were separated with SDS-PAGE. Gel bands corresponding to molecule weight (MW) 60 Kd were sliced off, processed into tryptic peptides, and analyzed by LC-MS/MS. *E*, representative quadrant plots demonstrate the impact of cofilin C139A mutation on ONE-induced(100 μM, 10 h) apoptosis. Apoptosis was quantified *via* Annexin V/PI staining kits with flow cytometric analysis. *F*, apoptotic phase distribution and relative quantitative results were determined by measuring differential fluorescence intensities in cells. Specifically, the apoptotic rate of each experimental group was normalized to its respective vehicle (DMSO) control. The relative apoptotic state was calculated as the ratio of the apoptotic proportion after ONE treatment to that after DMSO treatment within the same cell group. Experiments were performed in biological triplicate. Data are shown as mean ± SEM. *G*, summary of adduction events at ACTG_328 across various other ONE-derived modifications. For each modification type, the table shows the total number of spectra (Spectrum Count), the median extracted ion chromatogram (XIC) area, and the standard deviation (SD) of the XIC area. These metrics provide an overview of the abundance and variability of each adduction type detected at the site. Lipid peroxidation produces the reactive aldehyde 4-oxo-2-nonenal (ONE), which modifies proteins and contributes to chronic diseases. This study reveals that ONE directly crosslinks vicinal cysteine and lysine residues in cellular proteins *via* Michael addition and Paal-Knorr condensation. Using chemoproteomics, 165 pyrrole-based Cys-Lys crosslinks on 145 proteins were identified. Functional studies demonstrate that a crosslink between cofilin and actin partially mediates ONE-induced apoptosis. These findings uncover a previously unknown mode of protein crosslinking by an endogenous electrophile. DMSO, dimethyl sulfoxide; HNE, 4-hydroxy-2-nonenal; NAC, N-acetylcysteine; ONE, 4-oxo-2-nonenal.

Furthermore, we asked whether aONE-mediated crosslinks can exert biological effects by modulating protein–protein interactions. To this end, an interprotein crosslink between COF1 and the actin subunit ACTG immediately caught our attention, as COF1 has been demonstrated to play an important role in actin function (*i.e*., depolymerization or polymerization) and be closely associated with cancer and nervous system diseases (52). In a 3D structure of the COF1-ACTG complex, the αC-αC distance of the crosslinked cysteine and lysine is only 10.2 Å (Fig. 5*A*), which is in accordance with the fact that the crosslinked C139 site of COF1 is localized in the actin-binding domain (53). Using western blotting, we first confirmed the parent compound ONE indeed caused crosslinking within COF1-ACTG proteins in a concentration- or a time-dependent fashion (Fig. 5*B*), while another well-studied lipid electrophile 4-hydroxy-2-nonenal did not induce the formation of the COF1-ACTG complex (Fig. 5*C*). In addition, the COF1-ACTG complex crosslinked by ONE was tolerable to reducing reagent like N-acetylated cysteine (Fig. 5*C*), further ruling out the possibility of disulfide bond-based complex. Moreover, the MW 60 kDa-oriented in-gel digestion and subsequent MS analysis further verified the ONE-mediated crosslinking of interprotein Cys 139-Lys 328 pair within the endogenous COF1-ACTG complex (Fig. 5*D*).

**Figure S13:**
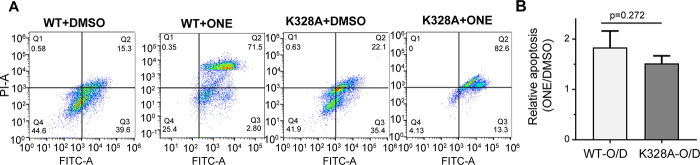


Of note, C139 of COF1 has been shown to be critical for maintaining its affinity for actin and oxidation of this site can cause COF1-dependent apoptosis by altering the permeability of the mitochondrial membrane and releasing cytochrome c (54). Hence, we asked whether ONE could induce apoptosis by mediating a crosslink *via* Cys139 of COF1 with K328 of Actin. To this end, HEK293T cells transiently transfected with WT and C139A mutant were treated with ONE and then analyzed with a flow cytometry-based apoptosis assay (Fig. 5*E*). Western blot analysis confirmed comparable expression levels of WT and C139A mutant constructs, and empty vector transfection did not induce apoptosis in the absence of ONE treatment (Supplemental Fig. S12*C*). To isolate the specific contribution of the crosslink from background effects (*e.g*., transfection or general ONE toxicity), we employed a relative quantification approach that normalizes responses within each experimental system. Statistical analysis (Fig. 5*F*) indicated that the C139 mutation universally attenuates cellular sensitivity to apoptosis induction across all experimental settings compared to the WT group. In contrast, the K328 mutation did not significantly reduce apoptosis sensitivity (Supplemental Fig. S13, *A* and *B*). This difference may be attributed to the fact that ACTG K328 can undergo various other ONE-derived modifications in addition to crosslinking (Fig. 5*G*, Supplementary Table S4).

Taken together, these results suggest that ONE-induced apoptosis in cells may be partially attributed to the crosslinking event between COF1 and ACTG. However, it is important to stress that ONE’s proapoptotic effects are not limited to a single mechanism. As a potent lipid-derived electrophile, ONE can modify numerous nucleophilic sites across the proteome, thereby affecting multiple signaling pathways. For example, ONE has been reported to activate the p53 pathway (55), induce mitochondrial dysfunction (8), and modify key regulatory proteins involved in cell survival and death.

## Discussion

Understanding the adduction chemistry of lipid-derived electrophiles is critical for elucidating their biological functions and mechanisms of action in cells. Since its discovery, ONE-mediated protein modifications have been extensively studied both *in vitro* and *in situ*. Although the potential of this bifunctional electrophile for crosslinking proteins has been recognized for a long time, little is known regarding whether and how it crosslinks cellular proteins, especially in living cells.

Here we provide the first survey of ONE-mediated *in situ* crosslinks at the proteome-wide scale using its ‘clickable’ surrogate. The analysis not only reveals the pyrrole-based vicinal Cys-Lys crosslink as a major chemotype from *in situ* ONE adduction, but also expands the inventory of ONE adducts by identifying a total of 165 crosslinks on 145 cellular proteins. The data adds further complexity to the target promiscuity of ONE, thereby laying the groundwork for further elucidating its mechanisms of action which remain an enigma. In fact, numerous crosslinked sites were previously determined to be functionally important, for which, as an initial attempt, we highlight the potential crosstalk between the ONE crosslinking of Cys-Lys pairs and cysteine-mediated redox regulation. Furthermore, our quantitative analysis offers an opportunity to compare *in situ* dynamics of ONE-mediated crosslinks with its initial modifications.

From the perspective of methodology, our work, together with several recent reports (56, 57, 58), highlights that chemoproteomics is particularly useful for the identification of potential crosslinking events induced by endogenous, highly electrophilic aldehyde metabolites. Here we adopt both affinity-based and activity-based chemoproteomic approaches, which not only provide a cross-validation, but also result in the discovery of a small number of crosslinks with relatively high site occupancy. Nonetheless, it is important to stress that exogenously applying ONE (for activity-based chemoproteomics) or aONE (for affinity-based chemoproteomics) to cells may not reflect cellular factors that modulate the formation and targets of endogenous ONE. For instance, ONE might accumulate at the endoplasmic reticulum membrane that has been demonstrated to be the main site of lipid peroxidation (59), which could not be mimicked by the present experimental settings for chemoproteomic profiling. Identifying the protein crosslinks and/or modifications by endogenous ONE, especially in certain cellular compartments, therefore remains to be an unmet technical challenge, as do other electrophilic metabolites. A related challenge concerns the measurement of endogenous ONE concentrations. Due to its high reactivity, ONE is rapidly consumed upon formation through covalent modification or crosslinking of proteins; thus, free steady-state concentrations are likely to be extremely low and may not reflect the true extent of exposure or the local concentrations at sites of production. This complexity is a recognized challenge in the field of reactive electrophile biology and underscores the need for contextual interpretation of exogenous treatment concentrations used in mechanistic studies. In addition, we acknowledge that the current profiling depth is limited, and direct quantitative comparison between crosslinked and mono-adducted peptides is challenging. Crosslinked species often exhibit distinct ionization and detection efficiencies compared to their mono-adducted counterparts, leading to inherent differences in the concentration-to-signal relationship between these two classes of peptides. Therefore, the relative abundances of crosslinked *versus* mono-adducted events should be interpreted with caution.

More generally, another interesting pursuit in the future is to harness the alpha, beta-unsaturated aldehyde group from ONE as a “zero-length” crosslinker to study vicinal Cys-Lys pairs. Historically, a number of vicinal Cys-Lys pairs have been demonstrated to be critical for the functions of certain proteins, such as biotin carboxylase Cys230-Lys238 (60), phosphoenolpyruvate carboxykinase Cys31-Lys39 (61), GSH transferase P1-1 Cys47-Lys54 (62) and rhodopsin Cys316-Lys325 (63). More recently, Tittmann and coworker discovered a novel, widespread redox switch that occurs in vicinal Cys-Lys pairs in proteins with a covalent nitrogen-oxygen-sulfur bridge (64, 65). These findings therefore necessitate the development of tools for probing vicinal Cys-Lys pairs at the proteome-wide scale. In order to target such a task, it is possible to generate tailored probes based upon aONE, for example, by shortening the carbon chain of the latter.

In summary, the identification of a pyrrole-based Cys-Lys crosslink by ONE *in situ* not only provides an additional insight into mechanisms of action of this bifunctional lipid-derived electrophile, but also offers an opportunity for developing bioinspired probes for XL-MS analysis.

## Data availability

The MS data sets have been deposited to the ProteomeXchange Consortium *via* the iProx partner repository with the dataset identifier PXD041968 (https://www.iprox.cn/).

## Supplementary data

The online version contains supplementary material available.

Correspondence and requests for materials should be addressed to Jing Yang (yangjing@ncpsb.org.cn) (15).

## Conflict of interest

The authors declare no competing interests.

## Figures and Tables

**Figure S1:**
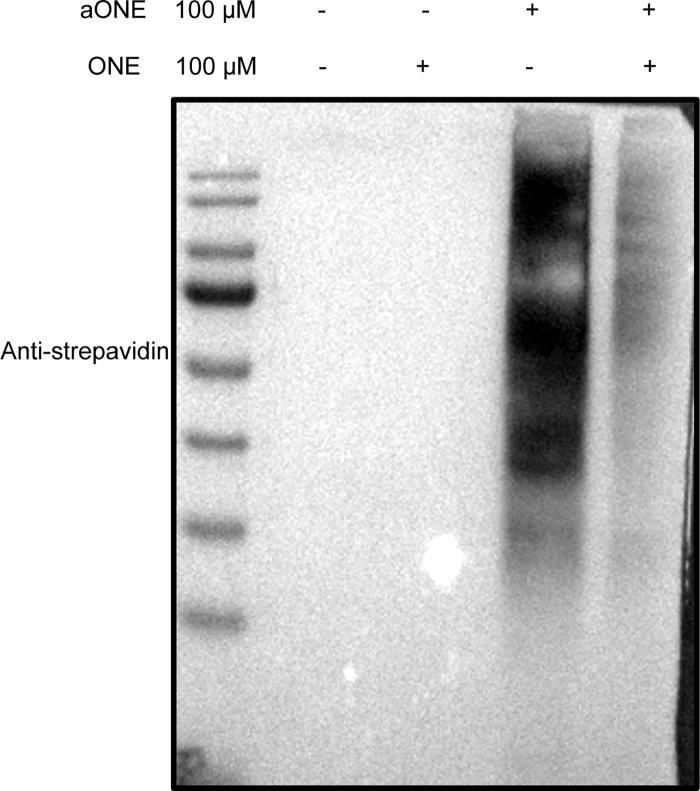


**Figure S14:**
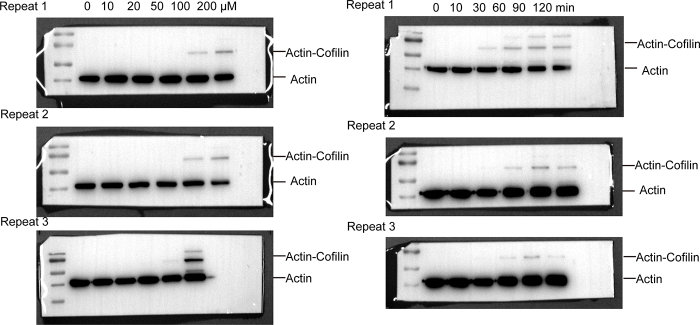


**Figure S15:**